# Is a focus on ‘recycling’ useful? A wider look at metal mutability and the chemical character of copper alloys

**DOI:** 10.1111/arcm.12753

**Published:** 2022-01-30

**Authors:** Peter Bray

**Affiliations:** ^1^ Department of Archaeology University of Reading Reading UK

**Keywords:** affordance, characterisation, chemical analysis, copper alloy, provenance, recycling, statistical analysis

## Abstract

Recycling is increasingly visible in archaeological descriptions of technology. This has a range of benefits as we attempt to engage with the full complexity of the material past. However, this paper examines in more detail whether a singular focus on recycling is positive for archaeological science. It considers the historical relationship between recycling and the chemical analysis of early copper alloys, the constraints of the different conceptual and statistical techniques that can be employed, and broader ideas of metal mutability and the characterisation hypothesis. Overall, anything that widens our conceptual toolkit should be welcomed. However, it is most helpful to have recycling emerge from case study data as part of a broader approach, rather than to focus on it as a sole aim. Similarly, the prioritisation of provenance has sometimes not helped the field. Engaging with a wider ‘characterisation hypothesis’ would bring several benefits, and help build collaborations with other areas of archaeology.

## INTRODUCTION

Recycling is on the rise in archaeological descriptions. This can be connected with the impact of environmental campaigning and concerns of sustainability. It is also a natural consequence of efforts to write complex artefact biographies, and the appreciation of the interplay of people and things. Debates over systematics and the creation processes of the archaeological record have always been concerned with recycling’s power to move material between contexts. All of these influences have long roots, and there are now several recent collections on recycling, as well as papers that focus upon key case studies or themes (Amick, 2015; Armada & García‐Vuelta, 2021; Bourgarit & Thomas, 2012; Bradley, 1988; Bray, 2020; Bray & Pollard, 2012; Caple, 2010; Degryse et al., 2006; Duckworth & Wilson, 2020; Duckworth et al., 2016; Fleming, 2012; Freestone, 2015; Gheorghiu & Mason, 2017; Karageorghis & Kassianidou, 1999; Needham, 1998, 2001; Park et al., 2021; Ponting & Levene, 2015; Sainsbury et al., 2021; Swift, 2012; this special issue).

The obvious answer to the question posed in the title is ‘Yes, of course!’ By allowing recycling in its explanatory models, archaeology can have a more realistic insight into production rates, chronology, social and economic relationships with materials, and the complex lifetimes of objects and their constituent matter. Recycling does not have to be present, and should not be assumed, but considering the possibility opens up a more nuanced past—and, frankly a more realistic past than echoing modernist tropes of consumption and single‐use objects, as captured in the infamous 1955 *Life* magazine article ‘Throwaway Living’ (Anonymous, 1955).

Clearly, recycling is now firmly on the agenda and that has bought hard‐won benefits to our study of ancient technology. I want to stress that breaking open new conceptual doors and identifying technological processes is not straightforward. However, this paper aims to explore in more detail how we can consistently study recycling (or its absence) in a key material, copper alloys, and ask whether focusing on ‘recycling’ itself as a research agenda will continue to be useful.

## RECYCLING IN THE HISTORY OF ARCHAEOMETALLURGY

The scientific study of archaeological copper alloys does not have an easy relationship with concepts of recycling. The history of the discipline shows that worries over chemical variability, metal mixing and reuse have affected research agendas. Early applications of chemical analysis made clear that the provenance of the copper was a primary concern. The Ancient Mining and Metallurgy Committee of the Royal Anthropological Institute (formed in 1945 as the Early Mining and Metallurgy Group (Anonymous, 1946)) began the first systematic chemical analyses of early British and Irish copper alloy artefacts (Benton et al., 1952). This was inspired by the work of Otto and Witter (1952) and others in Europe, and explicitly aimed to ‘determine chemically the precise sources of the metal used in this country’ (Benton et al., 1952, p. 89). Similarly, Earle Caley (1948), when discussing the principal applications of chemistry to archaeology, includes ‘the identification of materials’ and ‘indications as to sources of materials, the existence of commerce in particular materials, and the direction of trade routes’. However, Caley does not mention recycling or mixing in these sections.

Although the classic formulation of the ‘provenance hypothesis’ has always cautioned that any mixing or recycling has to be accounted for (Wilson & Pollard, 2001), it is hard to escape that feeling that not determining a solo provenance for an artefact has often been taken as a ‘failure’. Even when recycling was commonly discussed as a factor in the creation of the archaeological record (Bradley, 1988; Needham 1998, 2001; Schiffer, 1972) and is ubiquitous in the life cycle diagrams of materials (Ottaway, 2001), it was rarely engaged with scientifically. This was particularly true for the earliest periods of metallurgy, before the appearance of large deposits that were clearly scrap from approximately the European Middle Bronze Age onwards. Therefore, Hartmann and Sangmeister (1972, p. 625) see the reuse of bronze as a later development:

new varieties of copper dating from about 1,600 BC and thereafter can be legitimately suspected to have been manufactured from previously worked material. Hoarded older material—belonging to scrap collectors—features far more often in archaeological finds from this time than deposits of bars.

Similarly, in his strident defence of provenance Pernicka (2014, p. 257) allows for recycling, but for later, clear‐cut cases of scrap, such as Late Bronze Age bun‐shaped ingots containing semi‐melted pieces of identifiable recycled objects. Overall, he argues that

The question of recycling is so obvious because we are living in an era where the resources of some metals are becoming scarce and recycling is an economic necessity. (Pernicka, 2014, p. 258).

In their review of the application of lead isotopes to copper alloys, Stos‐Gale and Gale (2009, p. 195) explicitly argue that the lack of indisputable provenance signals in chemical analyses of copper alloys, and the variety within that data, meant the approach had ‘failed’. For them, the perceived failure of chemical work spurred the development of lead isotopes ratio analysis of ores and artefacts.

This brief background perhaps explains that, while recycling is ubiquitous in schematic diagrams of formation processes or material life cycles, it remains controversial within archaeological science. Ponting and Levene (2015), for example, say that ‘Recycling makes things complicated for archaeometallurgists and archaeologists alike’; more recently, Ponting (2020, p. 265) calls recycling the ‘elephant in the room’, which has the awkward potential for adding uncertainty. Merkel (1982) uses the term ‘confusion’ for how remelting and chemical change can affect interpretations, while Melheim (2015) says that recycling appears to be a refuge, even excuse, when a chemical provenance cannot be established. Arguing that

It is my impression that recycling is sometimes introduced rather freely to account for irregular compositions—more out of convenience than actual knowledge of the particular metallurgical cycle.

Similar to the conclusions of Stos‐Gale and Gale (2009), Melheim (2015) states:

Clearly, element composition studies have failed in leading archaeologists to the mines.

There is a consistent theme that the provenance of the metal is the defining role of chemical analysis of copper alloys. Some have argued that this view has hampered parts of our discipline; for example, Craddock (1985, p. 59) says that

From the end of the Bronze Age the problems of multiplicity of sources and mixing of scrap metal have been deemed too daunting for [scientific] study and consequently relatively few analyses have been published from which information on the alloying practice might be obtained.

This, in part, also echoes Pernicka’s, and Hartmann and Sangmeister’s argument that recycling tends to be a later period problem.

Within the history of archaeometallurgy, recycling has been put in opposition to provenance several times. A further consequence of this separation is conceptual models which see ‘fresh metal’ and ‘recycled metal’ as mutually exclusive processes that dominate in a given time or place. To quote Pernicka (2014, p. 258) again:

Generally, one can assume that in expanding economies, the recycling rate should be small, because fresh metal must come into the system … On the other hand, in declining cultures, the recycling rate should increase, because the economic structures become obsolete or are destroyed so the metal supply is interrupted.

One of the key shifts in wider scholarship on recycling’s role in the past it that it is no longer seen as monolithic processes. It cannot be contained within one arrow on a flow diagram or seen as a tap to be turned on when the (uniform) metal supply is dimmed.

## SEEING RECYCLING AND THE WIDER CHEMICAL CHARACTER

We now have an exciting range of case studies, review volumes and papers that capture a wider world of material use and reuse. Several themes emerge which help flesh out the complexity of past engagements with metal, including written accounts of recycling and the importance of scrap in ancient processes (Bourgarit & Thomas, 2012; Craddock, 2016); corpora of objects found ‘out of their time’, which show the potential for curation, reclamation and reimagining earlier artefacts (Adkins & Adkins, 1985; Dark, 1993; Eckardt, 2004; Ferris & Smith, 1995; Hingley, 2009; scrap and half molten objects in ingots, production sites and transport contexts (Armada & García‐Vuelta, 2021; Valentza, 2018); and close analysis of typo‐chronology which can show local casting in non‐metal producing areas (Needham, Leese, Hook, & Hughes, 1989). In the remainder of this paper, I want to focus on what consistent chemical tools may assist these wider debates about copper alloy mutability, and return to the question of whether pursuing recycling directly is useful.

As well as discussing recycling and the diversity of technological processes in the past, an important connected debate is to critically reappraise our data analysis tools. What do different chemical, conceptual and statistical techniques allow us to see, and are some closing off lines of investigation? What tools do we have and what processes are they adept at identifying? I would argue that we do not actually have many *direct* chemical techniques to identify recycling; instead, we have broader signs of change and alteration which have to be constrained by further arguments.

We obviously have a number of pragmatic factors to juggle when collecting analytical data: precision, accuracy, detection limits, timescale, expense, volume and sampling method. Beyond that we also have to debate whether we use open or bounded chemical classifications; what statistical systems are applied; whether we use top‐down definitions of process or bottom‐up data‐led interpretations; whether we believe in laws of geo‐chemical process or contextual inferences; and how we can identify scales of practice ranging from individual objects, to assemblages and further. Each of these decisions affords us different degrees of constraint in identifying recycling and other processes. I would argue that the contentious history of recycling in chemical archaeometallurgy summarised above has often been affected by the interpretative techniques that have been used—not just the experience of the scientists.

Using other terms, this has been discussed for a long time. Needham, for example, argues against the use of cluster analysis for two reasons (Needham et al., 1989): first, it leads to the proliferation of slightly different classification schemes, which makes broader comparisons and links extremely difficult (also discussed by Blanchet et al., 1985); secondly, Needham argues that choosing cluster analysis makes it extremely difficult to see recycling or chemical ‘fuzziness’ as it does not readily allow analysts to see overlaps or blending of identified ‘groups’. Choosing cluster analysis means that a certain pool of explanations of the raw data has already been chosen.

A type of cluster analysis was applied by the *Studien zu den Anfängen der Metallurgie* (SAM group; Hartmann & Sangmeister, 1972; Junghans et al., 1960, 1968). This was built on the belief that the distribution of elements in the Earth’s crust followed a log‐normal distribution pattern (Vistelius, 1960), which would pass into similar repeating patterns representing workshops or centres of metallurgical production (Hartmann & Sangmeister, 1972). Therefore, this bounded chemical approach expected to see groups, based upon fundamental geochemical regularities, both of which make recycling hard to accommodate into any interpretations. The log‐frequency distribution of the elements is still widely cited today (Pernicka, 1999, 2014; Rademakers et al., 2020, p. 15). It would appear to give a direct and independent way to identify some technological processes—one that can cut through some of the complexity of human behaviour. However, the work of Reimann and Filzmoser (2000) directly tested the fundamental law of geochemical processes on a wide range of case studies, and concluded it was a myth. Geochemical data do not confirm to either normal or log‐normal distributions. They conclude:

Methods requiring a multivariate normal distribution are especially vulnerable when used with geochemical and environmental data and will often deliver unstable and faulty results. Geochemists and environmental scientists should realise that in very many cases they are actually presenting biased and faulty results by still believing in the lognormal law of distribution of their data. It is high time that they stop to uncritically use techniques that were not made for such situations. (Reimann & Filzmoser, 2000, p. 1014)

They go on to recommend that simple graphical visualisations of chemical distributions will give more important geochemical insights than very advanced statistical methods.

Stos‐Gale and Gale employ a different style of statistical analysis when using lead isotope ratio data to link artefact results to ore values. This begins with finding close matches in three‐dimensional space between the artefact and underlying ore fields. Then, a series of checks are performed to ensure that this is a reasonable provenance: comparing the isotope distribution of the artefact assemblage to that of the matched ore field; and looking at the geochemical information and archaeological evidence for the exploitation of those mines (Ling et al., 2014; Stos‐Gale & Gale, 2009). This prioritises a provenance match as the standard result, which then has to be excluded by further checks. A great deal of attention is paid in their work to showing that lead isotopes do not fractionate during smelting or casting, as this would move the artefact signal away from the ore source. Recycling as a potential explanation of the final isotopic ratio in the artefact is very briefly mentioned, and does not receive a numbered section in the methodology (Stos‐Gale & Gale, 2009, p. 205). Again, it is clear that the statistical choices made by the scientists make some modes of interpretation easier to arrive at than others. In the case of Ling et al., 2014, their conclusions on metal provenance have received some criticism for seeing unlikely matches far away from Scandinavia—for example, Cyprus (Radivojević et al., 2018). More recent work has demonstrated that the source of the metal is probably closer to the deposition point, with the signals altered through mixing (Nørgaard et al., 2019).

Even if we are cautious with our approach to scientific data on archaeological metals, and try to capture all the variation that is possible within the word ‘recycling’, we can still get bogged down in definitions. Schiffer (1972) describes simple flow models for both durable and consumable elements of the material assemblage. He separates ‘recycling’, where the object is routed back into the manufacture of a new object, and ‘lateral recycling’, where after the end of an object’s use‐life it is moved on to other activities, often after some maintenance, storage or movement. However, as Schiffer (1972, p. 159) immediately admits:

I wish to emphasize that these models are only simplifications of a stubbornly complex reality. They are not likely to fit neatly the sequences of activities in which elements of all cultural systems participate within their systemic contexts.

We can play with further top‐down definitions of key recycling operations, by including a variety of solid and liquid transformation processes, highlight the separation or mixing of units of material and so on. When I discussed a possible copper alloy recycling categorisation (Bray, 2020, fig. 7.3), it was more to show how quickly it becomes unwieldy and unusable. Very quickly you arrive at (at least) eight terms beginning with ‘re‐’: reprocessing, reworking, reforming, remodelling, recycling–consolidation, recycling–division, recycling, reclamation. These terms have little explanatory power on their own, and still do not capture all the various ways metal objects can be interacted with and modified. Even worse, we do not want to be locked into defining certain processes as ‘real recycling’, when what we are interested in are all past technological actions and their social meaning.

## AN OPEN APPROACH TO COPPER ALLOY MUTABILITY (NOT JUST RECYCLING)

Overall, based on these examples and the broader history of research, we can perhaps begin to recommend some ways forward for interpreting scientific datasets for copper alloys. Over several case studies, we have aimed to explore how the chemical dataset for copper alloys acts as a palimpsest. There is an underlying signal from ores and smelting practice, which can be then overwritten, pulled and twisted by later mixing, working, recycling and object use. Importantly, old object to new object recycling is not the only way that chemical composition can shift (Bray, 2016, 2020; Bray & Pollard, 2012; Bray et al., 2015; Cuénod et al., 2015; Hsu et al., 2016; Sainsbury et al., 2021). If we use the analogy of archaeological dating methods, traditional provenance chemical interpretations claim to produce ‘absolute dates’—a signal that could be interpreted as a unique geographical location. What is more realistic is a *relative* chemical approach, in which there may be consistent order and structure caused by metallurgical and social processes, but these have to be interpreted in context. The matching, clustering or grouping methods critiqued above often rely on the assumptions of ‘absolute chemistry’, which is being undercut by new research on underlying geochemical patterns (Reimann & Filzmoser, 2000), and the appreciation that units of material in the past had complex lives.

Rather than assuming that there are clusters, specific ranges or any other structure within the data, an open visualisation approach has been developed by myself and applied in collaboration with several colleagues (Bray, 2015; Cuénod et al., 2015; Hsu et al., 2016; Liu et al., 2015; Perucchetti et al., 2015; Pollard et al., 2017). The underlying concept is to define a chemical space, into which metal chemistry assemblages can be placed, to see how they may fall together or apart, trend or even space out chaotically (Bray et al., 2015). The ‘copper space’ is often defined in our work by arsenic, antimony, silver and nickel. Other important elements can be included in the concept, particularly iron, bismuth, cobalt, manganese, sulphur and gold. ‘Assemblage’ may refer to any archaeologically relevant grouping, so units that have been employed include geographical areas, artefact typologies, time periods, distance from mines or production areas, worked and unworked casts and so on.

Defining an alloying chemical space, impurity chemical space, and then looking for trends within those elemental combinations allows us to thoroughly explore trends and structures in the data. Importantly, it does this *without* making any prior assumptions about possible clusters, distribution shapes or fingerprints. It allows the relationships within the data to guide interpretation, rather than using top‐down models. This approach makes it feasible to see unaltered metal use, recycling, down‐the‐line remelting, and other processes in context and through integration with all other lines of archaeological evidence. It is important to highlight that one of the first applications of the approach—interpreting chemical analysis of the first metalwork in Britain and Ireland—showed that *c*. 2300 bc (Metalwork Assemblage 1 and 2; Needham et al., 1989) copper axes were moving unaltered from Ireland into Britain (Bray & Pollard, 2012). This is not an approach that is looking for recycling; instead, it aims to highlight the chemical character of the assemblage being studied.

One key tool in this approach is to look for systematic chemical changes within the trace elements in the copper. These have long been a practical industrial concern in the modern day; for example, the Institute of Foundrymen set up a committee to track metal losses and chemical alteration in mid‐20th‐century factories (Hampton et al., 1965, p. 225). There now exists a substantial literature on the general process of how remelting and casting copper alloys, even in slightly oxidising conditions, can alter the overall chemical character of an assemblage (Budd, 1991; Budd & Ottaway, 1991; Charles, 1980; Godfrey, 1996; McKerrell & Tylecote, 1972; Pickles, 1998; Sabatini, 2015). These include loss of vulnerable oxides of arsenic and antimony, and also segregation effects which would lead to later change through mechanical abrasion of enriched surfaces. We can also demonstrate the blending of chemical character through mixing—for example, the deviation away from the ingot signal into complicated recycled artefact assemblages (Bray, 2016, 2020). The concerns that these processes would obscure useful provenance patterns (McKerrell & Tylecote, 1972; Merkel, 1982; Tylecote, 1970) could be replaced by the attitude that they instead add great value to the chemical datasets, as processes can print other aspects of the history of a unit of copper alloy onto the chemical datasets (Bray & Pollard, 2012; Bray et al., 2015).

However, understanding these chemical trends is not easy; they are rarely an absolute or independent proxy for a technological process. To give one example, within the British and Irish Early Bronze Age Needham et al. (1989) argued that casting of new local object types in non‐metalliferous regions demonstrated a reliance on recycling metal from elsewhere. We can chemically support that argument by showing the progressive, step‐wise loss of arsenic away from a maximum level near the mine—Ross Island, County Kerry, Ireland (O’Brien, 2004)—to lower levels in Scotland, and then lower still in southern and eastern England (Bray & Pollard, 2012). This follows the initial movement of unaltered copper axes that can be chemically demonstrated for the preceding period (Metalwork Assemblage 1 and 2; Needham et al., 1989). As familiarity with metal grew and knowledge spread, the chemical data support a model of down‐the‐line metal exchange in Metalwork Assemblage 3 (*c*. 2,200–1900 bc; Needham et al., 1989). Now, each region begins to recycling their neighbour’s objects and casts them into locally approved forms, leading to small losses and other chemical changes during casting. However, strictly speaking, this chemical pattern is not directly showing recycling—just a shift downwards which requires further context to explain. At other points in history this chemical effect was deliberately used in order to refine copper alloys.

Klein and von Kaenel discuss the methods available to Romans for producing high‐purity copper, including oxygen refining (Klein & von Kaenel, 2000, p. 87). It is clear that Pliny recognised that copper from various regions had different properties due to impurities (Craddock, 2016, p. 205), and that processing could produce a standard product:

for all copper, after impurities have been rather carefully removed by fire and melted out of it, becomes *regulare*. (*Natural History* 34:94, translation quoted by Craddock, 2016, p. 205)

Therefore, the same chemical effect (a small loss of arsenic and antimony) that was incidental in the British and Irish Early Bronze Age was being deliberately used in Roman Italy. Only by context and the triangulation of other forms of evidence can we see one as recycling and one as deliberate refining. What is common, however, is the usefulness of open, graphical, data‐led systems of interpretation which help highlight copper alloy mutability.

## CONCLUSION: THE CHARACTERISATION HYPOTHESIS

In conclusion, a focus on recycling is helpful as it expands our understanding of complex technological and economic practice, social relationships with things, and also the formation of the archaeological record. However, for copper alloys at least, there are few chemical tools that can *directly* indicate recycling. Instead, what we are left with are complex palimpsests of several factors that created chemical structures and then altered them. Several of these ‘altering processes’ are covered by the banner recycling, but several others are not: such as refining, surface treatments, changes in smelting technology, different ore treatments including roasting, alloying, deliberate fragmentation before deposition, and sharpening. Overall, an exclusive focus that prioritises one aspect of metal technology to explain the chemical character of ancient metal is severely limiting, whether that is recycling, provenance or production technology.

A more positive way forward is to be critical about what our scientific tools and concepts afford us, work to overcome their shortcomings and build on their strengths, in order to understand all levels of copper alloy’s chemical character. I think this is best done using an open, graphical, universally applicable approach to chemical interpretation. There are obviously many statistical and visualisation choices at hand and, again, exploring a variety of these helps the field. Whatever the method employed, we should aim to avoid each research project producing detailed, but incompatible chemical groupings, that are then hard to repurpose to understand wider, nuanced, flows of material.

Context, collaboration and co‐operation are essential. Within a broad formulation of the characterisation hypothesis—that we can reconstruct the full lifetimes of ancient units of metal—scientific information is just one of many sources of information. Even if we can demonstrate repeated shifts in chemical character that indicate recycling of metals, that needs to be seen with other archaeological datasets to pin down intent, social impact and value. Finally, the only way we can approach this is through bringing together large volumes of data and recycling them (Huggett, 2018). A huge strength of scientific approaches to the past is that the chemical data can be reused and reimagined endlessly (Bray et al., 2015). Returning to Craddock’s argument (1985) that recycling had prevented the analysis of later period assemblages, hopefully an interest in recycling within archaeology will encourage new large‐scale analytical projects to join the remarkable achievements of previous generations of archaeological scientists.

## Data Availability

Data sharing is not applicable to this article as no new data were created or analyzed in this study.
